# TLR3 is required for survival following *Coxsackievirus* B3 infection by driving T lymphocyte activation and polarization: The role of dendritic cells

**DOI:** 10.1371/journal.pone.0185819

**Published:** 2017-10-03

**Authors:** Renata Sesti-Costa, Marcela Cristina Santiago Françozo, Grace Kelly Silva, José Luiz Proenca-Modena, João Santana Silva

**Affiliations:** 1 Department of Biochemistry and Immunology, School of Medicine of Ribeirão Preto, University of São Paulo, Ribeirão Preto, SP, Brazil; 2 Institute of Infection Immunology, TWINCORE, Centre for Experimental and Clinical Infection Research GmbH, Hannover, Germany; 3 Department of Genetics, Evolution and Bioagents, Institute of Biology, University of Campinas (UNICAMP), Campinas, Brazil; University of British Columbia, CANADA

## Abstract

Type B coxsackievirus (CVB) is a common cause of acute and chronic myocarditis, meningitis and pancreatitis, often leading to heart failure and pancreatic deficiency. The polarization of CD4^+^ T lymphocytes and their cytokine milieu are key factors in the outcome of CVB-induced diseases. Thus, sensing the virus and driving the adaptive immune response are essential for the establishment of a protective immune response. TLR3 is a crucial virus recognition receptor that confers the host with resistance to CVB infection. In the current study, we found that TLR3 expression in dendritic cells plays a role in their activation upon CVB3 infection in vitro, as TLR3-deficient dendritic cells up-regulate CD80 and CD86 to a less degree than WT cells. Instead, they up-regulated the inhibitory molecule PD-L1 and secreted considerably lower levels of TNF-α and IL-10 and a higher level of IL-23. T lymphocyte proliferation in co-culture with CVB3-infected dendritic cells was increased by TLR3-expressing DCs and other cells. Furthermore, in the absence of TLR3, the T lymphocyte response was shifted toward a Th17 profile, which was previously reported to be deleterious for the host. TLR3-deficient mice were very susceptible to CVB3 infection, with increased pancreatic injury and extensive inflammatory infiltrate in the heart that was associated with uncontrolled viral replication. Adoptive transfer of TLR3^+^ dendritic cells slightly improved the survival of TLR-deficient mice following CVB3 infection. Therefore, our findings highlight the importance of TLR3 signaling in DCs and in other cells to induce activation and polarization of the CD4^+^ T lymphocyte response toward a Th1 profile and consequently for a better outcome of CVB3 infection. These data provide new insight into the immune-mediated mechanisms by which CVBs are recognized and cleared in order to prevent the development of myocarditis and pancreatitis and may contribute to the design of therapies for enteroviral infections.

## Introduction

Type B coxsackieviruses (CVBs) are single-stranded (+)-sense RNA viruses that belong to the enterovirus genus and *Picornaviridae* family. They are highly cytolytic and can induce a wide range of acute and chronic diseases, such as myocarditis, meningitis and pancreatitis, with some serotypes being associated with the development of diabetes [1–4]. With a prevalence of 45% amongst infants and adolescents with acute myocarditis or dilated cardiomyopathy, CVB infection is considered one of the most common causes of infectious myocarditis [5–7].

The clinical course of the diseases caused by CVB varies from limited cardiac and pancreatic acinar damage to heart failure and pancreatic deficiency, with increasing patient morbidity and mortality [8]. CVB persistence has been related to chronic cardiomyopathy and pancreatitis [9], indicating that the virus is able to evade immune surveillance in some circumstances. Accordingly, CVB3 infection causes chronic myocarditis in some mouse strains, such as A.BY/SnJ, A/J and SWR/J [10], whereas the virus is controlled during the acute phase in other strains, such as 129Sv/J and C57BL/6 [10–12].

In resistant strains, CVB3-induced myocarditis and pancreatitis are abrogated by T lymphocyte-dependent mechanisms, despite the fact that CVB can evade CD8^+^ T cell responses by reducing its presentation on MHC-I (major histocompatibility complex) [13]. CD4^+^ T cell priming and polarization as well as the balance of adaptive cytokines are key factors in the outcome of CVB-induced disease. Although the Th1 response contributes to some degree of tissue damage [14], interferon (IFN)-γ-producing Th1 cells are responsible for controlling CVB and resolving the infection [15]. In contrast, a Th17-skewed immune response is harmful to the host, as it is unable to control viral replication and contributes to tissue damage, worsening the CVB infection scenario [16,17]. Th2 cytokines are associated with both improvement [18,19] and aggravation [20] of CVB-induced disease. The intensity of the response and the microenvironment in which these cytokines are produced might influence the balance of the immune response and the outcome of infection. Indeed, a Th1 response together with the presence of regulatory T cells in target tissues is crucial in providing the regulation necessary for preventing tissue injury [21–23].

The priming and polarization of T lymphocyte responses depend on viral recognition by antigen-presenting cells. Viral RNA is sensed by toll-like receptors (TLRs) and RIG-I-like receptors (RLRs). RIG-I and MDA-5 are cytosolic helicases that detect distinct forms of dsRNA that accumulate in the cytosol upon viral replication. Their expression is induced by type I interferon (IFN-I) in most cell types [24]. Among the TLRs, TLR3 and TLR7/8 recognize dsRNA and ssRNA, respectively. Viral RNA becomes accessible to these receptors in the endosome either upon phagocytosis of infected apoptotic cells, the internalization of immune complexes with viral RNA, or by autophagy of cytoplasmic RNA. Although some stromal cells express RNA-recognizing TLRs, these receptors are mainly expressed by antigen-presenting cells, especially dendritic cells (DCs) and macrophages [25,26].

TLRs activate overlapping but different signaling pathways upon stimulation. The intracellular adaptor protein MyD88, which is activated by all TLRs except TLR3, worsens CVB3-induced cardiac injury [27]. In contrast, TLR3- and TLR4-dependent TRIF signaling is essential for the antiviral response and has been implicated in the improvement of the disease [28,29]. TLR3-TRIF signaling results in the phosphorylation and nuclear translocation of the transcription factors IRF3 and NF-κB, which are crucial for the production of type I IFN and proinflammatory cytokines, respectively [30,31].

Innate immunity against CVB is dependent on TLR3, as TLR3-deficient mice develop fulminant myocarditis and are unable to sustain high levels of IFN-I [11]. In addition, the polarization of adaptive immunity is also affected by TLR3, as it has been shown that TLR3-deficient mice on the B6.129 background develop a IL-4-producing Th2 response profile during CVB3 myocarditis instead of the IFN-γ-producing Th1 phenotype observed in resistant B6.129 mice [28]. Furthermore, studies using human biopsies associate polymorphisms in *tlr3* gene with increased incidence of enteroviral myocarditis [32], indicating a role for TLR3 in the resolution of enteroviral infections.

TLR3 signaling in macrophages is important for survival following CVB4 infection [33], though the mechanisms underlying this effect are still unclear. However, whether the expression of TLR3 by DCs plays a role in the immunity against CVB infection has yet to be determined. DCs are important cells that bridge the innate and adaptive immune responses against viral infections. They are specialized in presenting antigen to T lymphocytes, thus promoting their activation and polarization [34]. CVB3 infection was found to promote the generation of DCs from bone marrow and their migration to the myocardium [35]. DCs were shown to be activated upon CVB3-induced myocarditis, resulting in IFN-γ stimulation and IL-2 production by CD8^+^ T lymphocytes [36]. In addition, CVB3-resistant C57BL/6 DCs secrete higher levels of IL-6, TNF-α and IL-10 than do permissive A.BY/SnJ cells [11]. These findings indicate that DCs play a critical role in the immune response against CVB and prompted us to question whether the expression of TLR3 by DCs plays a role in the outcome of CVB3 infection.

We found that TLR3-deficient DCs were less efficient in activating T lymphocytes upon CVB3 infection, as they exhibited reduced up-regulation of co-stimulatory molecules. They up-regulated the inhibitory molecule PD-L1 and secreted less TNF-α and IL-10 and more IL-23. The induction of T lymphocyte proliferation by CVB3-infected DCs was decreased in the absence of TLR3, and the lymphocyte response was skewed toward a Th17 profile. TLR3-deficient mice were very susceptible to CVB3 infection, with extensive inflammatory cell infiltration in the heart that was associated with uncontrolled viral replication or virus persistence. Adoptive transfer of TLR3^+^ DCs to TLR3-deficient mice slightly increased their survival following CVB3 infection. Thus, our findings show the role of TLR3-expressing DCs in the activation and polarization of the CD4^+^ T lymphocyte response and indicate that they play a role together with other TLR3^+^ cells on the resolution of CVB3 infection, as these cells impair the development of myocarditis and pancreatitis.

## Results

### TLR3 affects the activation of and cytokine production by CVB3-infected dendritic cells

To investigate the role of DC-expressed TLR3 during CVB infection, we first characterized the activation markers and cytokine production controlled by TLR signaling in bone marrow-derived DCs infected with CVB3 in culture. Co-stimulatory and inhibitory molecules, together with secreted cytokines, orchestrate the activation and polarization of lymphocytes toward different profiles.

**Fig 1:**
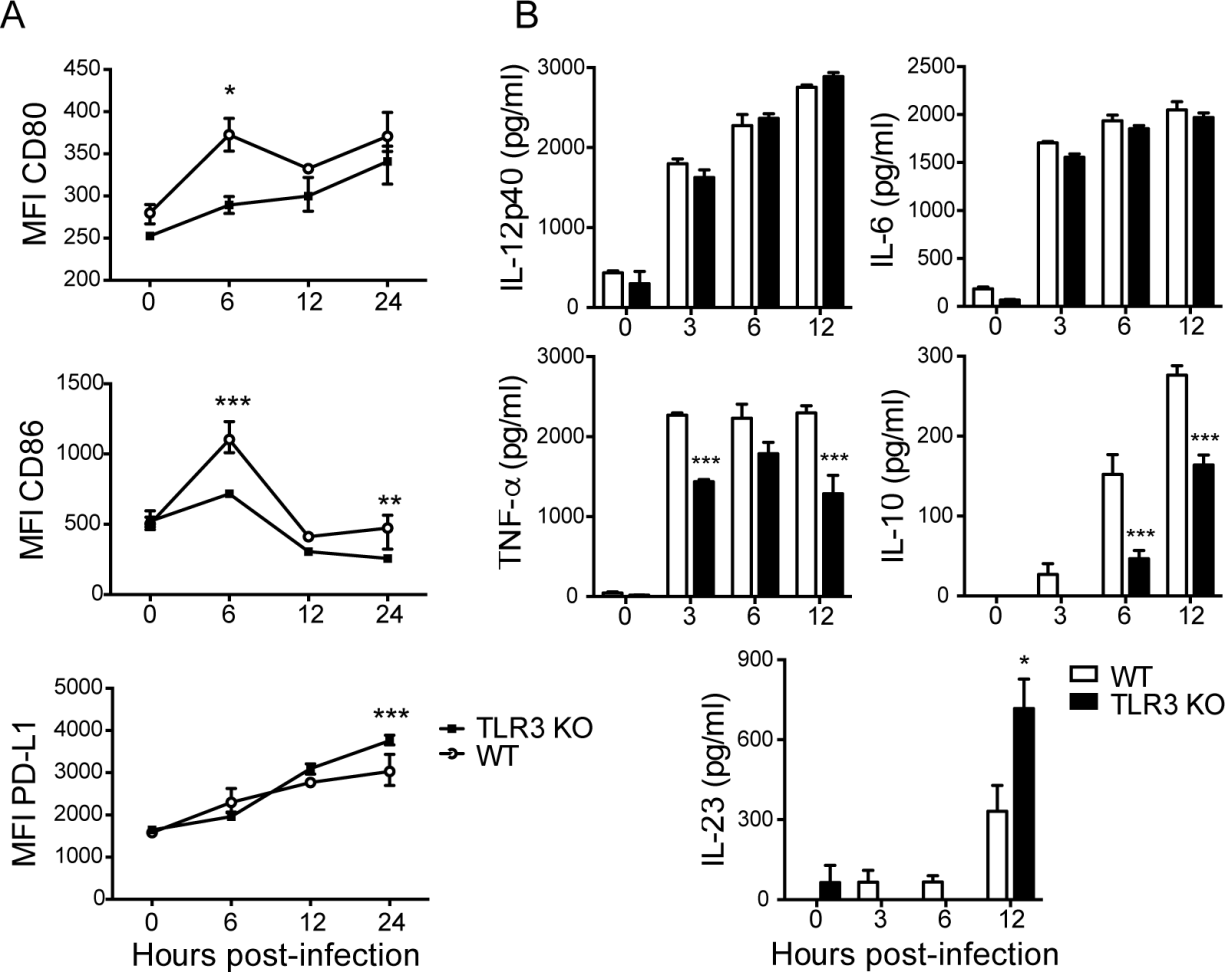
CVB3-induced activation and cytokine production by dendritic cells are modulated by TLR3. DCs were differentiated in culture from bone marrow cells with 20 ng/ml GM-CSF for 7 days. Cells were infected with CVB3 (MOI: 10) for the indicated time. (A) Expression of CD80, CD86 and PD-L1 was assessed by flow cytometry, and (B) cytokine levels in the supernatant were measured by ELISA. MFI: Median fluorescence intensity. Data are means of n = 3 + SE for each time point, and are representative of two independent experiments. Two-way ANOVA followed by Bonferroni’s test was used for all comparisons. P<0.001: ***; P<0.01:**; and P<0.05:*.

Whereas previous reports, analyzing late points after infection, were not able to show activation by DCs upon CVB3 [13,36], our findings show that WT DCs up-regulate CD80 and CD86 as early as 6 hours after CVB3 infection. Furthermore, TLR3 seems to affect antigen presentation and co-stimulation by DCs after CVB3 infection, as TLR3-deficient DCs exhibited reduced expression of those markers. Moreover, in the absence of TLR3, DCs expressed higher levels of the inhibitory molecule PD-L1 later in culture (24 hpi) (Fig 1A and S1 Fig). The production of some cytokines by DCs infected with CVB3 was also dependent on TLR3. TLR3-deficient DCs produced strikingly less TNF-α and IL-10 and exhibited a slight increase in IL-23 secretion 12 hours after infection. The production of IL-6 and IL-12p40 was not affected by TLR3 deficiency (Fig 1B).

These data show that the expression of TLR3 by DCS is important for CVB3 RNA recognition and indicate that the presentation of viral peptides and the co-stimulation of T lymphocytes might be impaired in the absence of TLR3. In addition, polarization of the T-cell response might be affected by the altered cytokine production by DCs.

### TLR3 is essential for T lymphocyte proliferation and shifts their polarization after CVB3 infection

**Fig 2:**
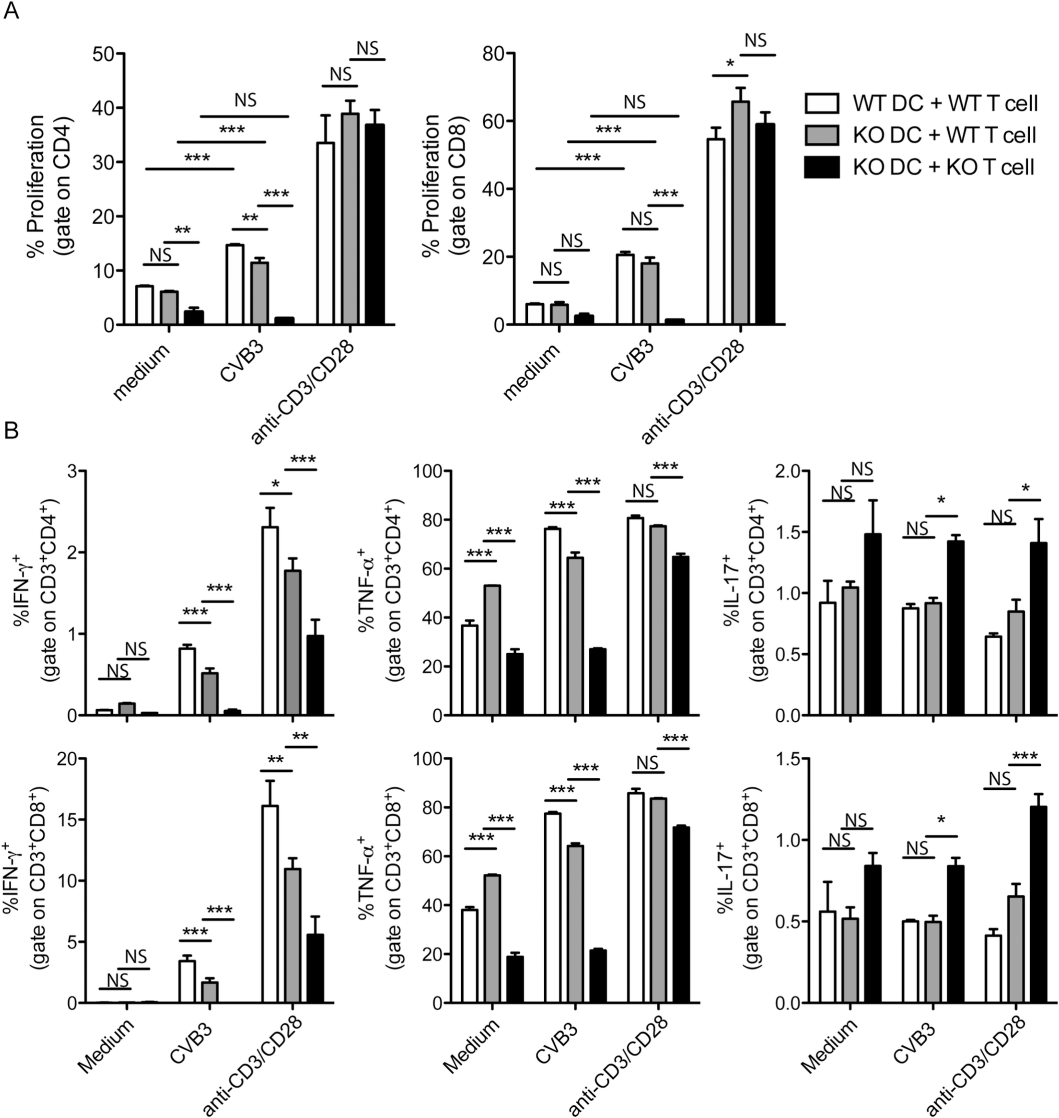
TLR3 modulates the proliferation and cytokine production of CD4^+^ and CD8^+^ T lymphocytes after stimulation with CVB3-infected dendritic cells. WT and TLR3 KO DCs were differentiated in culture from bone marrow cells with 20 ng/ml GM-CSF for 7 days. Cells were infected with CVB3 (MOI: 10) or incubated with medium and co-cultured with isolated WT or TLR3 KO T lymphocytes from CVB3-infected mice (5 dpi) at a ratio of 10 lymphocytes/1 DC. In some wells, anti-CD3 and anti-CD28 were added for the polyclonal stimulation of lymphocytes. (A) Lymphocytes were stained with CFSE, and proliferation was assessed after 3 days of co-culture via CFSE dilution in the CD4^+^ or CD8^+^ populations. (B) Intracellular cytokines were measured by flow cytometry after re-stimulation with PMA and ionomycin in the presence of Golgi Stop. DC: dendritic cells; T: T lymphocytes. Data are means of n = 3 + SE (3 uninfected DCs and T cells from 3 infected mice) for each condition (medium, CVB3 and anti-CD3 + anti-CD28) and are representative of two independent experiments. Two-way ANOVA followed by Bonferroni’s test was used for all comparisons. P<0.001: ***; P<0.01:**; and P<0.05:*; NS: non-significant. All analyzed parameters in CVB3-infected condition are significantly different between WT DC + WT T cell vs KO DC + KO T cell.

To test the hypothesis that TLR3-deficient DCs might affect the T lymphocyte response, we co-cultured CVB3-infected DCs with T lymphocytes isolated from CVB3-infected mice. To evaluate T lymphocyte activation, cells were stained with CFSE, and proliferation was assessed by CFSE dilution after 3 days of co-culture. WT DCs induced the proliferation of both CD4^+^ and CD8^+^ T lymphocytes; however, the proliferation of both CD4^+^ and CD8^+^ T cells was almost completely abolished when the T cells were isolated from infected TLR3-deficient mice, whereas proliferation of CD4^+^ T cells was slightly decreased when DC were deficient in TLR3. When anti-CD3 and anti-CD28 antibodies were added to the culture, T lymphocyte proliferation was restored, indicating no problem with T cell proliferative capacity and that the modulation is DC and CVB3-dependent (Fig 2A and S2 Fig).

The cytokines produced by T lymphocytes in co-culture were also affected by TLR3. CD4^+^ and CD8^+^ T lymphocytes co-cultured with TLR3-deficient DCs produced less IFN-γ and TNF-α than those co-cultured with WT DCs. When both DCs and T lymphocytes were isolated from TLR3 KO mice, the percentage (Fig 2B and S3 Fig) and total number (S4 Fig) of cells producing these cytokines was even lower. Although there was no difference in IL-17 production by T lymphocytes when they were co-cultured with TLR3-deficient DCs, TLR3-deficient lymphocytes produced higher levels of IL-17 than WT lymphocytes. This phenomenon was observed for both CD4^+^ and CD8^+^ T lymphocytes. These cytokine changes also occurred when anti-CD3 and anti-CD28 antibodies were added to the culture (Fig 2B and S3 and S4 Figs).

Taken together, these data show that TLR3-expressing DCs, together with other TLR3-expressing cells, play a role on polarization toward a Th1 profile. Interestingly, TLR3 diminishes the development of a Th17 response in a DCs-independent way. Because all these changes in proliferation and cytokines production by T lymphocytes were observed when T lymphocytes were isolated from infected TLR3 KO mice, cells other than DCs might be responsible for the effects.

### TLR3 affects dendritic cell activation and T lymphocyte polarization after in vivo infection with CVB3

We next investigated whether the phenomenon described in vitro is reproduced in vivo. For this purpose, WT and TLR3 KO mice were intraperitoneally (i.p.) infected with 10^6^ CVB3 for 3 or 7 days, after which DC activation markers in the spleen and the mediastinal and pancreatic lymph nodes were observed, as CVB has been shown to infect and replicate in the heart and pancreas of humans and mice. Cytokine production by T lymphocytes was also investigated after 3, 7 and 12 days of infection.

**Fig 3:**
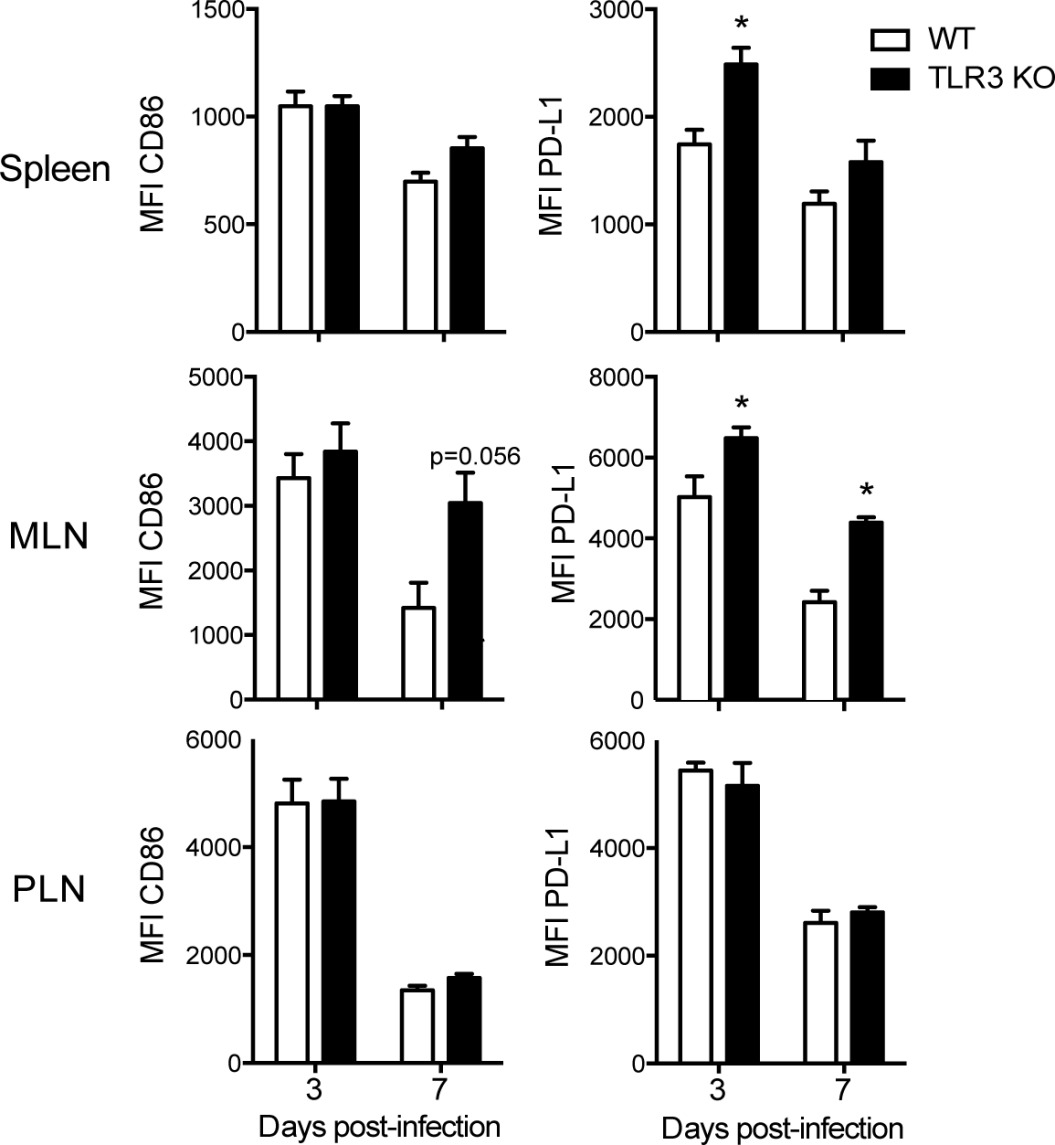
Dendritic cell activation after in vivo CVB3 infection is altered by TLR3. WT and TLR3 KO mice were i.p. infected with 10^6^ CVB3. Cells from the spleen and the mediastinal (MLN) and pancreatic (PLN) lymph nodes were isolated at the indicated times, and activation markers were evaluated by flow cytometry. MFI: Median fluorescence intensity. The MFI was measured in the CD11c^+^ population. Data are means of n = 3–5 + SE for each group and each time point, and are representative of three independent experiments. Two-way ANOVA followed by Bonferroni’s test was used for all comparisons. P<0.05:*.

**Fig 4:**
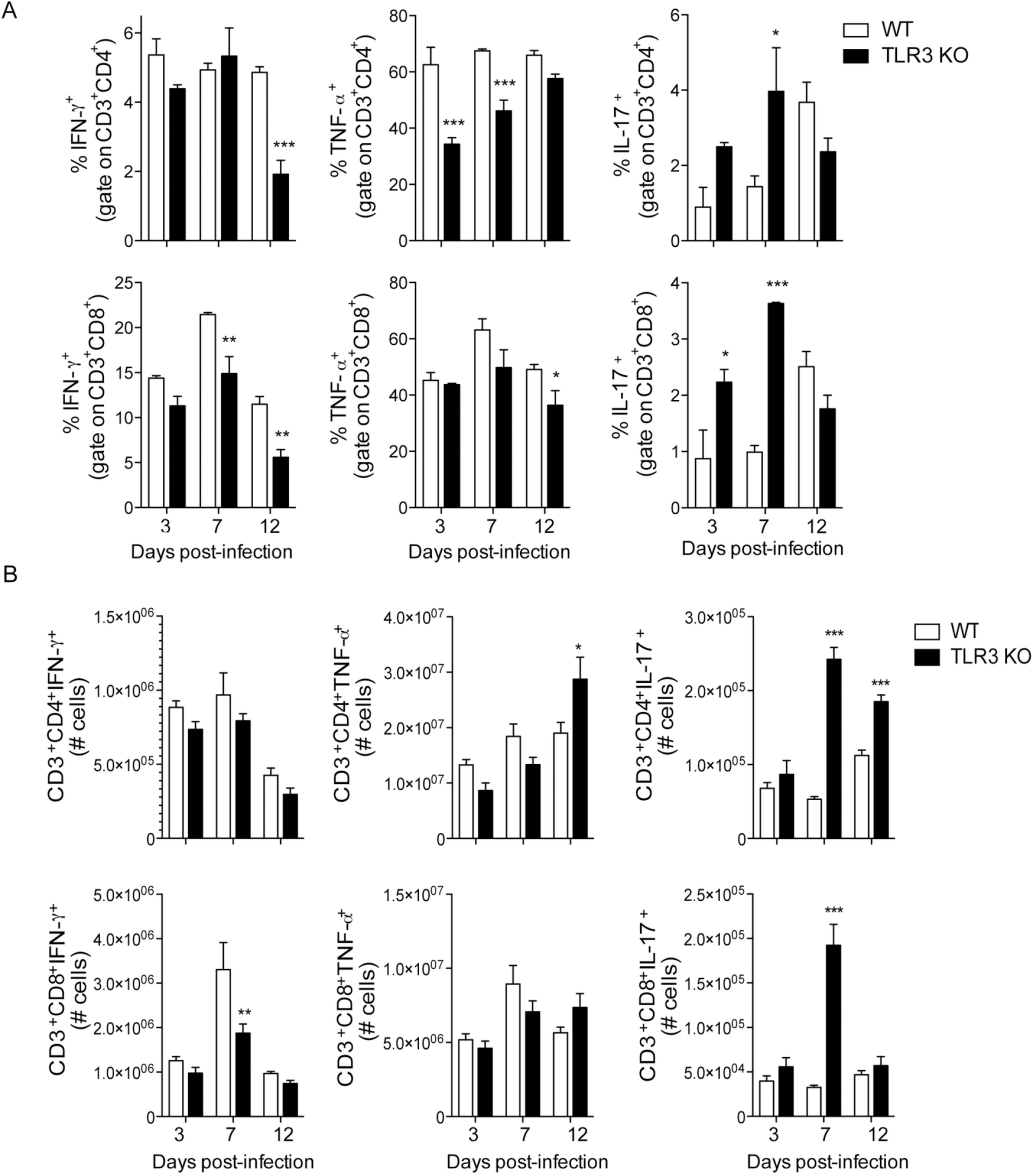
TLR3 skews the T lymphocyte response towards Th1 and inhibits the Th17 profile after CVB3 infection. WT and TLR3 KO mice were i.p. infected with 10^6^ CVB3. Spleen cells were isolated at the indicated times and stimulated with PMA and ionomycin in the presence of Golgi Stop for 6 h. Intracellular cytokines were measured by flow cytometry. (A) Percentage and (B) Total number of cytokines-producing T cells/spleen. Data are means of n = 4–5 + SE for each group and each time point, and are representative of three independent experiments. Two-way ANOVA followed by Bonferroni’s test was used for all comparisons. P<0.001:***; P<0.01:**; and P<0.05:*.

In accordance with the in vitro data, DCs from the spleen and mediastinal lymph nodes of infected TLR3 KO mice exhibited increased expression of PD-L1, although no change was seen in CD86 expression. There was no change in the expression of these molecules in DCs from pancreatic lymph nodes (Fig 3 and S5 Fig). Furthermore, TLR3 KO mice have lower percentage and absolute number of IFN-γ and TNF-α-producing CD4^+^ and CD8^+^ T lymphocytes than WT mice in the spleen, whereas the IL-17-producing T cells were higher. (Fig 4 and S6 Fig). These data corroborate the findings from the in vitro experiments and suggest that TLR3 is important for the development of a Th1 response and the inhibition of a Th17 response after infection with CVB3.

**Fig 5:**
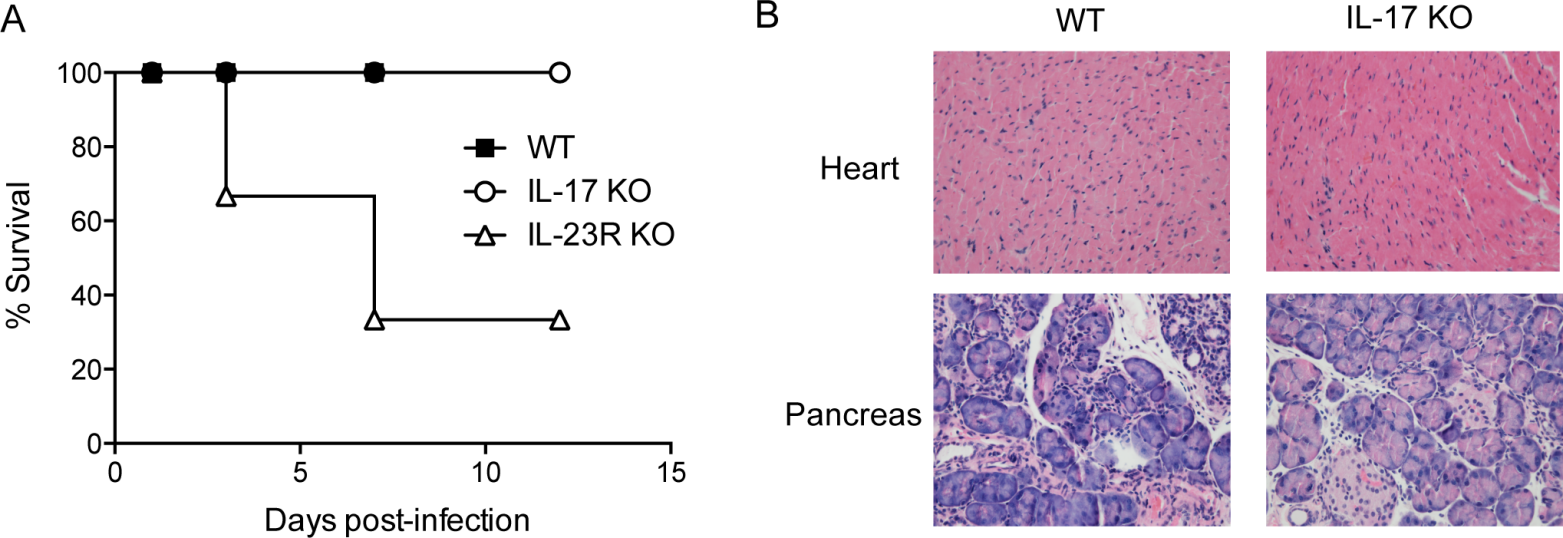
Mice deficient in IL-17 are resistant to CVB3 infection. WT, IL-17 KO and IL-23R KO mice were i.p. infected with 10^6^ CVB3. (A) Survival was monitored. (B) Heart (top panel) and pancreas (bottom panel) sections were stained with hematoxylin and eosin at 12 dpi. Data are means of n = 5 + SE for each group, and are representative of two independent experiments. Gehan-Breslow-Wilcoxon test was used to compare survival curves, which showed p = 0.0175 for WT vs IL-23R KO.

It has been previously demonstrated that a Th1 response is important for the clearance of CVB infection [15], whereas a Th17-biased profile of immune response is detrimental to the host, as it exacerbates inflammation that is not capable of eliminating the virus, thus compromising tissue function [16]. Corroborating these findings, IL-17 KO mice survived infection with 10^6^ CVB3, similar to WT mice (Fig 5A). The pancreases of IL-17 KO mice exhibited inflammatory infiltrate and edema similar to those in WT mice, whereas the hearts of neither mouse strain were affected by the virus after 12 days of infection (Fig 5B and S7 Fig). The cytokine IL-23 has been shown to be important for maintenance of Th17 lymphocytes. Since, TLR3-deficient DCs produced more IL-23 upon CVB3 infection in vitro, we wondered whether it could be responsible for the shift on T cell response. Mice deficient in the IL-23 receptor, nonetheless, were extremely susceptible to infection, as some of them died as early as 3 days after infection with 10^6^ CVB3 (Fig 5A). These data suggest that IL-23 might play an important role in CVB infection, other than its function in promoting and maintaining the development of Th17 cells.

The results show that TLR3 is an important receptor in the recognition of CVB3 in vivo. The absence of TLR3 impairs DC activation and the differentiation of T lymphocytes towards Th1 cells, increasing the differentiation of Th17 lymphocytes. The lack of IL-17, in turn, does not induce susceptibility to CVB3 infection.

### TLR3-expressing cells confer resistance to CVB3 infection

**Fig 6:**
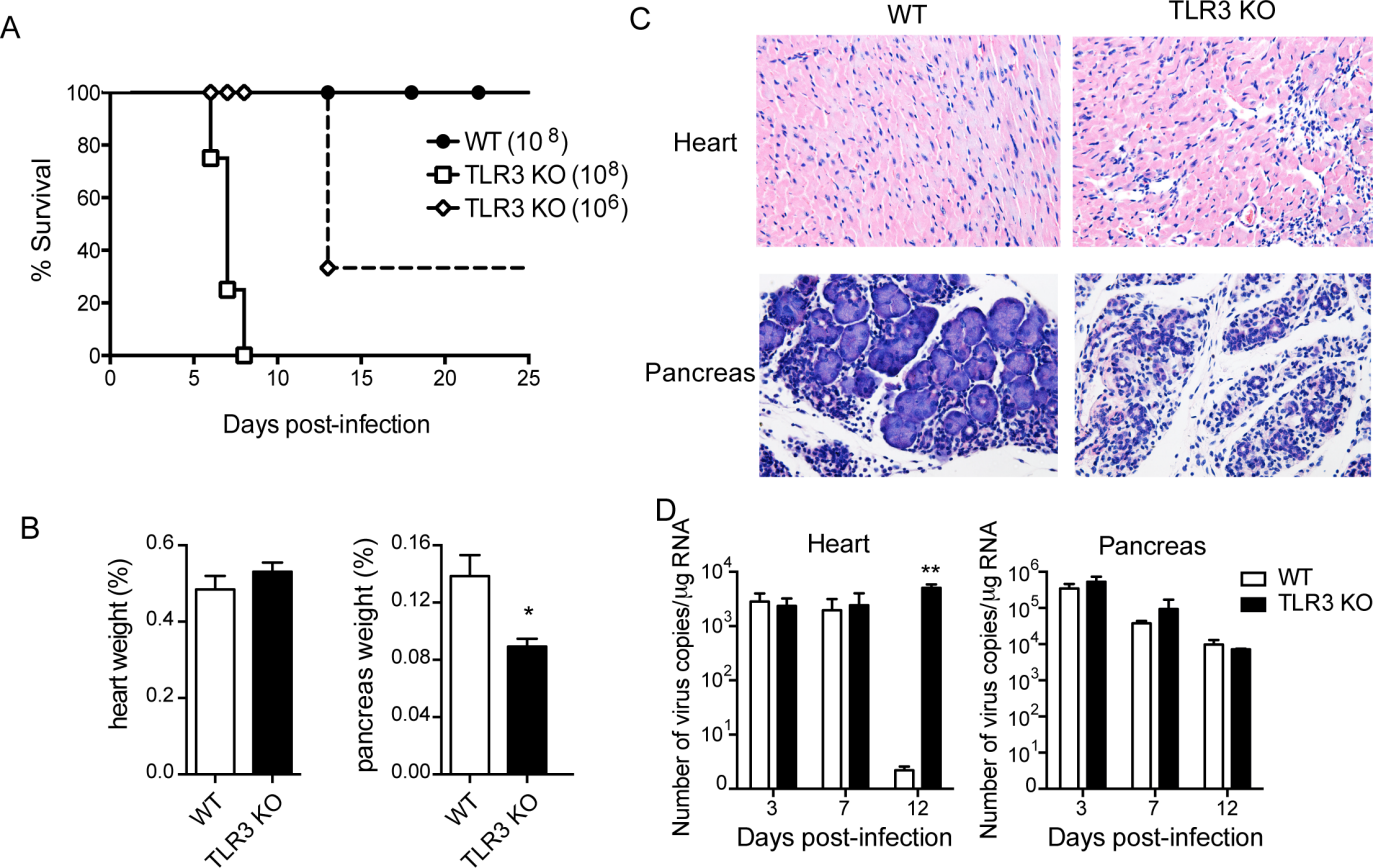
TLR3 is essential for controlling tissue inflammation and viral load after CVB3 infection. WT and TLR3 KO mice were i.p. infected with 10^8^ or 10^6^ CVB3. (A) Survival was monitored for 25 days. After infection with 10^6^ CVB3, (B) the weights of the heart and pancreas at 12 dpi are shown as a percentage of body weight; (C) heart (top panel) and pancreas (bottom panel) sections were stained with hematoxylin and eosin; and (D) viral load was determined from PCR of the heart and pancreas at the indicated times. Data are expressed as number of virus copies/μg RNA. Data are means of n = 3–6 + SE for each group and each time point. Unpaired *Student t* test was used to compare tissue weight and Two-way ANOVA followed by Bonferroni’s test was used for all comparisons of viral load. P<0.01:**; and P<0.05:*. Gehan-Breslow-Wilcoxon test was used to compare survival curves, which showed p = 0.0006 for WT vs TLR3 KO (10^8^), and p = 0.03 for WT vs TLR3 KO (10^6^).

With the purpose of verifying the role of TLR3 in resistance to infection, we infected WT and TLR3 KO mice with CVB3 and monitored their survival. In accordance with previous data [37], WT mice were extremely resistant to infection with CVB3, as they survived inoculation with 10^8^ viruses. In contrast, TLR3 KO mice succumbed to infection with 10^8^ viruses in less than 8 days. The susceptibility of TLR3 KO mice was so extreme that only 35% of mice survived more than 13 days of infection with a 100-fold lower viral load (10^6^) (Fig 6A).

Although the heart weights of WT and TLR3 KO mice were similar after CVB3 infection, the hearts of TLR3 KO mice exhibited considerable inflammatory infiltrate 12 days post-infection that was not observed in hearts from WT mice (Fig 6B and 6C and S7 Fig). CVB3 infection also induced inflammation in the pancreases of both WT and KO mice. However, pancreases from TLR3 KO mice exhibited more severe edema and tissue damage, while pancreases from WT mice had some intact acinar structures 12 days post-infection (Fig 6C and S7 Fig). As a consequence, the pancreatic weights of TLR3 KO mice were significantly reduced at this time point (Fig 6B). After infection, a great amount of virus was found in the hearts and pancreases of both strains of mice. Although the viral load in the pancreas remained high after 12 days of infection, it progressively decreased over time in a similar manner in WT and TLR3 KO mice (Fig 6D), suggesting that differences on pancreas injury cannot be attributed to a difference on either virus replication or virus genome persistence in the tissue. The viral load in the hearts of WT mice was barely detected 12 days after infection. In contrast, the viral load in the hearts of TLR3 KO mice remained very high (Fig 6D), showing that TLR3 is necessary for controlling the virus replication or persistence in the heart.

**Fig 7:**
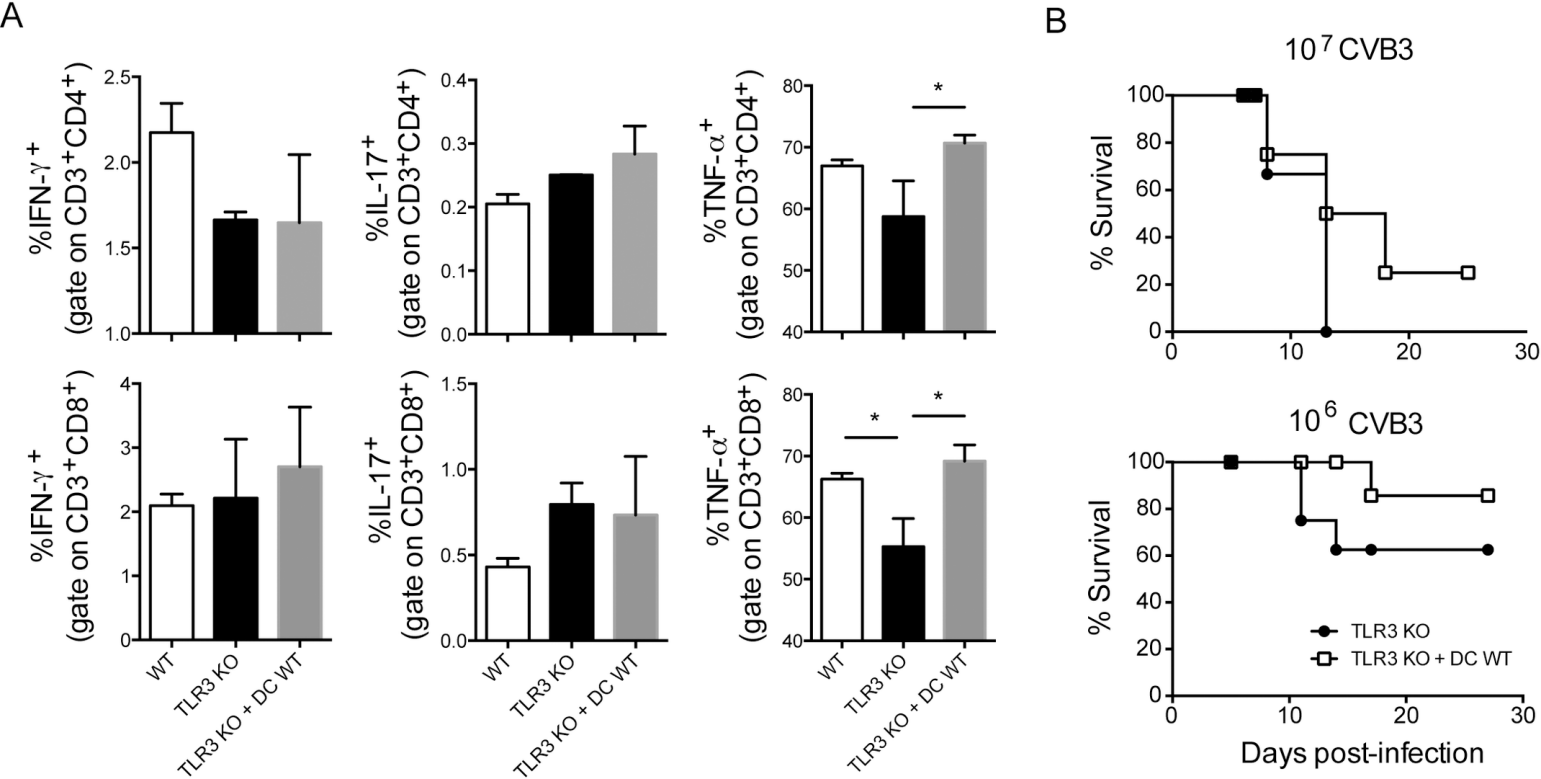
Adoptive transfer of TLR3^+^ dendritic cells increases TNF-α production by T lymphocytes and slightly improves the survival of CVB3-infected mice. WT and TLR3 KO mice were i.p. infected with either 10^7^ or 10^6^ CVB3. WT bone marrow-derived DCs (2 x 10^6^) were adoptively transferred i.v. to TLR3 KO mice 1 day post-infection. (A) At 12 dpi with 10^6^ CVB3, spleen cells were isolated and stimulated with PMA and ionomycin in the presence of Golgi Stop for 6 h. Intracellular cytokines were measured by flow cytometry. (B) Survival was monitored. Data are means of n = 3–6 + SE for each group, and are representative of three independent experiments. One-way ANOVA followed by Bonferroni’s test was used to compare the three groups in (A). *: p<0.05. Gehan-Breslow-Wilcoxon test showed no significant difference between the survival curves in (B).

Studies have indicated the role of DCs in the adaptive anti-viral response during CVB infection [38]. In addition, TLR3-expressing DCs are correlated with resistance to CVB-induced myocarditis [11]. To investigate the role of TLR3^+^ DCs in resistance to CVB3 infection, we differentiated DCs in culture from bone marrow cells of WT mice and adoptively transferred them to TLR3 KO mice 1 day after infection with either 10^7^ CVB3 (a viral load that kills all TLR3 KO mice) or 10^6^ CVB3 (a viral load that kills approximately 60% of TLR3 KO mice). Although the adoptive transfer of DCs did not change the production of IFN-γ and IL-17 by T lymphocytes, it did restore the percentage of TNF-α-producing CD4^+^ and CD8^+^ T lymphocytes, without changing the absolute number of cells (Fig 7A and S8 and S9 Figs). Moreover, their transfer slightly increased the survival of mice infected with either viral load (Fig 7B), indicating that TLR3^+^ cells other than DCs may play a major role on survival upon CVB3 infection. The increased survival after DCs transfer can also be due the increased number of DCs regardless of TLR3 expression.

Altogether, our data show that TLR3 is important for modulating DC activation and cytokine production during CVB3 infection in vitro. In vivo, the expression of TLR3 by other cell types, besides DCs may be responsible for inducing the proliferation and polarization of specific T lymphocytes toward the protective Th1 profile and for inhibiting the harmful Th17 response. As a consequence, TLR3 is essential for elimination of the virus in the heart and for tissue preservation.

## Discussion

Infection by CVB induces myocarditis and acute pancreatitis in humans and mice. CVB is a single-stranded RNA virus with positive polarity; thus, its genome acts as mRNA inside the cell and is translated by the cellular machinery. A double-stranded RNA intermediate is likely synthesized throughout viral replication, causing the viral genome to be recognized by TLR3 in the endosome and stimulate TRIF signaling [39–41]. Although it has previously been shown that CVB3 does not infect or replicate efficiently in DCs, positive and negative strand viral RNAs have been found in these cells [11], indicating that DCs might be directly activated by the virus. We confirmed that hypothesis in the present study, as we found that DCs up-regulated the co-stimulatory molecules CD80 and CD86, which contribute to the priming of naïve T cells, upon CVB3 infection. Although in vitro generation of CD11c^+^ DCs with GM-CSF from bone marrow is likely to contain other cell types, which might contribute to altered function, the data indicate that the expression of TLR3 by DCs contributed to their activation, as TLR3-deficient DCs show a less pronounced activated phenotype after CVB3 infection compared to WT DCs. Instead, they up-regulated the inhibitory molecule PD-L1, which inhibit T lymphocyte activation and proliferation by binding to PD-1.

In vivo, infecting TLR3 KO mice with CVB3 confirmed these data, as DCs from the spleen and mediastinal lymph nodes presented an inhibitory phenotype compared to WT cells, we speculate that viral dsRNA formed after CVB3 replication is recognized by TLR3 and, through TRIF signaling, results in changes on expression of antigen-presenting molecules, although we cannot exclude the possibilities that CVB3 differently infect TLR3 KO DCs and that infection of the host may also indirectly affect the functions of DCs. The phagocytosis of infected debris containing dsRNA and its internalization into DC endosomes might permit TLR3 stimulation in this scenario, as previously demonstrated [42].

The secretion of IP-10 and RANTES by CD4^-^CD8^+^ DCs, which express 10-fold more TLR3, is important in conferring resistance to CVB infection [11]. Our data show that TLR3 expression by DCs is important not only for chemokine secretion but also for other cytokines such as TNF-α and IL-10, which are produced at considerably lower levels in TLR3-deficient DCs, and IL-23, which was increased in these cells. Although TNF-α is associated with the aggravation of CVB3-induced myocarditis [12], it supports IFN signaling during viral infection [43], which is important for controlling viral replication. Thus, certain levels of this cytokine might contribute to defense against virus, causing minimal damage. IL-10 was previously shown to be cardioprotective due to its prevention of excessive inflammation [44]. Accordingly, regulatory T cells, which produce IL-10, attenuate CVB5-induced pancreatitis [23]. Thus, DC-derived IL-10 might contribute to the outcome of CVB-associated disease by preventing inflammation-induced tissue injury and necrosis. IL-23, on the other hand, is known to maintain the Th17 lymphocyte population [45], which contributes to the exacerbation of CVB-induced tissue injury [16].

When we investigated the ability of DCs to stimulate T lymphocytes, we found that TLR3-deficient DCs barely altered CD4^+^ T lymphocyte proliferation. When T lymphocytes were isolated from TLR3 KO mice, however, they were completely unable to proliferate in the presence of DCs and CVB3, which show that TLR3-expressing cells, other than DCs, play the role on stimulation of T cell proliferation. The levels of IFN-γ and TNF-α produced by lymphocytes, however, were reduced after stimulation with TLR3-deficient DCs and in TLR3 KO mice. The IL-17-producing lymphocyte population, on the other hand, was considerably higher in TLR3 KO-infected mice. Although TLR3-deficient DCs were not able to directly drive lymphocytes from WT mice to a Th17 profile in co-culture, the T lymphocytes isolated from TLR3 KO-infected mice produced higher levels of IL-17. Our co-culture experiments revealed that T cells isolated from infected TLR3 KO mice affected T cell proliferation and cytokines production even more than isolated TLR3 KO DCs did. Those T cells were primed by DCs in vivo. Besides, other TLR3-deficient-cells might play a role in this scenario, including T cells themselves. We speculate that DCs might act together with other cells to affect T cells response in TLR3 KO mice in vivo or that CVB3 might affect DCs both directly and indirectly. Indeed, TLR3 can recognize dsRNA in other hematopoietic cells or even stromal cells, inducing a rise in systemic IFN-I, which can activate DCs, thus improving their ability to prime and drive T lymphocyte responses [46].

Previous reports have found that IFN-γ is a crucial factor in preventing severe dilated cardiomyopathy and heart failure after CVB3 infection [47], and IFN-γ-producing CD8^+^ T cells are required to impair the development of chronic myocarditis [48]. The evidence generated thus far suggests that DCs play a role in this scenario, as they were shown to be activated in CVB3-induced myocarditis, resulting in the stimulation of IFN-γ and IL-2 production by CD8^+^ T lymphocytes [36]. We show in the current study that DCs are indeed directly involved in priming and driving T lymphocytes to become IFN-γ- and TNF-α-producing cells upon CVB3 infection and that the shift in T lymphocyte response is dependent on TLR3 expression in DCs. These data corroborate the results of some studies that have shown reduced production of IFN-γ after CVB3 infection in the absence of TLR3 and TRIF [28,29,37].

The susceptible A.BY/SnJ mouse strain exhibits a Th2-biased response with impaired IFN-γ production [11], indicating that a humoral immune response may predominate over a cellular response in susceptible animals. TLR3 deficiency has been shown to shift the T cell response toward a Th2 profile during CVB3 infection [28]. IL-4 production was associated with the induction of chronic inflammatory cardiomyopathy in TLR3 KO mice [20]. TRIF-deficient mice also develop a Th2-biased response with high production of IL-33 [28], which is nonetheless different from the response in TLR3-deficient mice, indicating that TLR3 acts together with TLR4, the other TLR that activates TRIF signaling, in the impairment of CVB3-induced disease. We found, for the first time, that there is a shift in T cell response to a Th17 profile in the lack of TLR3. In addition, our findings corroborate previous reports indicating that IL-17 is dispensable for the host following CVB3 infection [16,49,50], as IL-17 KO mice were resistant to infection and exhibited slightly reduced pancreatic injury compared with WT mice. However, IL-23R KO mice were strongly susceptible to CVB3 infection, suggesting that this cytokine plays an important role in preventing lethal CVB3-induced injury through mechanisms that are still unclear.

TLR3 has been shown to be dispensable or even harmful in several viral infections, such as infection with reovirus [51], influenza A [52], rabies virus [53], and West Nile virus (WNV) [54]. TLR3 seems to mediate the neuroinvasiveness of rabies virus and WNV, contributing to the lethal progression of these diseases. For other viruses, including picornaviruses such as encephalomyocarditis virus (EMCV) [55], poliovirus [56], and type B Coxsackievirus (CVB) [11,28,33,57], TLR3 is associated with the impairment of viral replication and control of tissue damage, resulting in a better outcome of the virus-induced diseases. In agreement with these findings, we observed that TLR3 KO mice are extremely susceptible to CVB3 infection, as they died even after infection with a viral concentration at least 2 logs lower than the concentration that C57BL/6 mice are able to resist. TLR3 KO mice were unable to control viral replication in the heart, and this effect was correlated with increased inflammatory infiltrate, suggesting that the presence of the virus continuously induces the migration of leukocytes to the tissue and that these cells mount an inappropriate response that is unable to kill the virus. TLR3 KO mice also exhibited greater necrosis in the pancreas, resulting in a decrease in pancreas weight.

TLRs are ubiquitously expressed in human and murine cells; however, they are expressed at the highest levels in immune cells, especially antigen-presenting cells [58]. The expression of TLR3 by macrophages is important in the resolution of CVB4 infection [33]. We now show the role of TLR3-expressing DCs in anti-CVB3 immune response, and indicate that other cells expressing TLR3 are also required to adaptive immune response against the virus and to improve the outcome of CVB3 infection.

DCs comprise different subsets of cells with some shared and other different functions. The subset that is responsible for TLR3-dependent recognition of CVB3 and priming of T lymphocytes remains unclear. Although plasmacytoid DCs are important cells in the response to viral infection [59], they do not express TLR3. CD8α^+^ conventional DCs are good candidates, as they are the cells that express more TLR3 among the distinct subsets [60]. On the other hand, the CD8α^-^ subset is more specialized in presenting antigen through MHC-II to CD4^+^ T cells [61] and could also be responsible for the TLR3-dependent skewing of the adaptive response towards Th1 that is observed during CVB3 infection.

CVB3 induces changes in the relative proportions of DC subsets and reduces the overall number of DCs. Inhibition of the CD8^+^ T cell response is associated with reduced numbers of CD8α^+^ DCs during CVB3 infection [38]. In addition, the resistant C57BL/6 mouse strain has a more rapid expansion and greater number of CD8α^+^ DCs in the spleen than the susceptible A.BY/SnJ strain upon CVB3 infection [11]. A recent study demonstrated that TLR3-mediated CD8α^+^ DC activation contributes to the antiviral state [62]. Whether CD8α^+^ DC is the subtype involved on impairment of CVB3 infection has yet to be determined.

In summary, the present study identified the role of TLR3-expressing DCs in driving the adaptive immune response toward a Th1 profile after CVB3 infection. This highlights the complexity of the host response to viral pathogens and the mechanism by which some receptors and adaptor molecules act in the balance of immunity. Our findings provide insight into the protective immune response during CVB3-induced myocarditis and pancreatitis and may contribute to the design of therapies against enteroviral infections.

## Materials and methods

### Mice and viral infection

CVB3 (Nancy strain—ATCC) was propagated in a Vero-cell monolayer in MEM (Life Technologies) supplemented with 2% FBS and antibiotics at 37°C. Titration was performed with limiting dilutions to determine the 50% cytopathic effect on the cellular monolayer (TCID_50_). Male C57BL/6, TLR3 KO, IL-17 KO and IL-23R KO mice (all in C57BL/6 background) were i.p. infected with 10^6^, 10^7^ or 10^8^ TCID_50_ CVB3 at age 5–7 weeks-old. Survival was monitored daily. The heart and pancreas were harvested on day 12, weighed, fixed in 10% formalin and embedded in paraffin. Slides of the tissues were stained with hematoxylin and eosin for examination with a light microscope. Lymph nodes and spleen were harvested on days 3, 7 and 12 for flow cytometry analysis.

### Ethics statement

This study was approved by the Ethical Commission of Ethics in Animal Research of School of Medicine of Ribeirão Preto, University of São Paulo, (protocol no. 029/2011). This commission is part of the National Brazilian College of Animal Experimentation. Anesthesia was performed with i.p injection of ketamine and xylazine before i.v. DCs transferring, and euthanasia was performed with the use of CO_2_ chamber. In animal survival experiments, we monitored mice survival daily and mice were euthanized with the use of CO_2_ chamber when they show any sign of suffering.

### Culture of bone marrow-derived dendritic cells

DCs were differentiated from bone marrow cells in culture by incubation in RPMI (Gibco BRL) supplemented with glutamine, penicillin, streptomycin and 5% fetal bovine serum, and containing 20 ng/ml GM-CSF (PeproTech) for 7 days. The medium was replenished on the third day. Differentiated DCs were infected with CVB3 (MOI: 10) for 1 hour, washed and incubated for 3, 6, 12 and 24 hours. The cells were analyzed by flow cytometry, and the cytokine levels in the supernatant were quantified. In some experiments, cells (2 x 10^6^) were adoptively transferred intravenously (i.v.) to mice after 1 day of CVB3 infection.

### Lymphocyte isolation and co-culture

Spleens from CVB3-infected mice (5 dpi) were harvested and minced to obtain a single-cell suspension. Red blood cells were lysed with lysis buffer (8.26 g/L NH_4_Cl; 1 g/L KHCO_3_; 0.037 g/L EDTA) for 5 min at room temperature. CD4^+^ and CD8^+^ T lymphocytes were sorted using anti-CD4 and anti-CD8 antibodies coupled to magnetic beads (Miltenyi Biotec) as described in the manufacturer’s instructions.

Isolated lymphocytes were co-cultured with either CVB3-infected bone marrow-derived DCs (10:1) or anti-CD3 (1 μg/ml) and anti-CD28 (2 μg/ml) antibodies for 24 h. Cells were re-stimulated with PMA (500 μg/ml) and ionomycin (50 μg/ml) in the presence of Golgi Stop (BD Biosciences) for 6 h, and cytokine production was evaluated by flow cytometry. To measure cell proliferation, sorted lymphocytes were stained with 2.5 μM CFSE for 5 min at room temperature. After being washed, the cells were co-cultured in 96-well plates with either CVB3-infected bone marrow-derived DCs or with anti-CD3 (1 μg/ml) and anti-CD28 (2 μg/ml) antibodies for 72 h. Lymphocyte proliferation was evaluated by flow cytometry based on CFSE dilution in the CD4 or CD8 gates. Percentage of proliferation was represented by the last rounds of proliferation based on anti-CD3/CD28 stimulation (S2A Fig).

### Flow cytometry

Bone marrow-derived DCs and single cell suspensions from the spleen and the mediastinal and pancreatic lymph nodes were evaluated for the expression of phenotypic markers. For this, cells were incubated with Fc block for 45 min to block Fc receptors. Monoclonal antibodies conjugated to PE, PE-Cy7, FITC, APC, APC-Cy7, Alexa Fluor-700 or PerCP were added and incubated for 30 min at 4°C. Antibodies against CD11c, CD80, CD86, and PD-L1 (BD Biosciences) were used to stain DCs. Cells were washed and evaluated by flow cytometry (FACSCanto BD).

To evaluate intracellular cytokines in lymphocytes, surface marker staining was performed as described above with antibodies against CD3, CD4 and CD8. Cells were washed, fixed and permeabilized using a Cytofix/Cytoperm kit (BD Biosciences) according to the manufacturer’s instructions. Cells were incubated with monoclonal antibodies against IFN-γ, TNF-α or IL-17 for 30 min at 4°C and evaluated by flow cytometry (FACSCanto BD). Data were analyzed using FlowJo (Tree Star).

### Detection of cytokines

The IL-12p40, IL-23, TNF-α, IL-6 and IL-10 levels in the DC culture supernatants were measured with an ELISA (BD Biosciences) according to the manufacturer’s instructions.

### Real time PCR

Total RNA was extracted from heart and pancreas using SV RNA isolation system (Promega). cDNAs were synthetized from RNA samples using High Capacity cDNA Reverse Transcription Kit (Applied Biosystems). Viral quantification was performed with Taqman PCR master mix and the following primers and probe for human enterovirus: HEV-F: 5’-GCGGAACCGACTACTTTGGG-3’, HEV-R: 5’-CTCAATTGTCACCATAAGCAGCC-3’, HEV-probe: Fam-TCCGTGTTTCCTTTTATTCTTATA-MGB (Applied Biosystems). For viral genome quantification, a 200-nt fragment from the 5’ untranslated region of CVB3 genome were cloned in a plasmid (pGEM®-T easy vector—Promega). The copy number of CVB3 genome was calculated based on a standard curve using a10-fold dilutions of this pGEM-HEV from 10^2^ to 10^8^ copies per reaction. Viral loads were determined as the copy number of viral genomes per μg of total RNA. The limit of detection of this assay was 2 copies of viral genome per reaction.

### Statistical analysis

Student *t* test was used for comparison of two experimental groups, and variance analysis (ANOVA) followed by Bonferroni’s test were used to determine differences among more than two groups. P value <0.05 was considered statistically significant. All error bars indicate standard error (SE).

## Supporting information

S1 Fig Activation of TLR3 KO DCs after in vitro infection with CVB3. Representative histograms from data presented on Fig 1A of CD80, CD86 and PD-L1 expression by DCs 0, 6, 12 and 24 hours post-infection with CVB3.
(TIF)

S2 Fig Proliferation of T lymphocytes stimulated with TLR3 KO DCs. (A) Representative histogram of proliferation of T lymphocytes stimulated with anti-CD3/CD28. Bars indicate different rounds of cell division. The last rounds of division represented percentage of proliferation. (B) Representative histograms from data presented on Fig 2A of CD4^+^ (top panel) and CD8^+^ (bottom panel) T lymphocytes proliferation stimulated with DCs in the presence of medium (left), CVB3 (center), or anti-CD3/CD28 (right).
(TIF)

S3 Fig Production of cytokines by T lymphocytes stimulated with TLR3 KO DCs. Representative dot plots from data presented on Fig 2B of production of IFN-γ (top panels), TNF-α (center panels), and IL-17 (bottom panels) by CD4^+^ and CD8^+^ T lymphocytes stimulated with DC in the presence of CVB3.
(TIF)

S4 Fig Number of IFN-γ-, TNF-α- and IL-17-producing T lymphocytes stimulated with TLR3 KO DCs. Absolute number of CD4^+^ and CD8^+^ T lymphocytes producing IFN-γ (left panels), TNF-α (center panels), and IL-17 (right panels) after stimulation with DC in the presence of medium, CVB3 or anti-CD3/CD28. All analyzed parameters in CVB3-infected condition are significantly different between WT DC + WT T cell vs KO DC + KO T cell.
(TIF)

S5 Fig Activation of TLR3 KO DCs after in vivo infection with CVB3. Representative histograms from data presented on Fig 3 of CD86 and PD-L1 expression by DCs on spleen, mediastinal (MLN) and pancreatic (PLN) lymph nodes 3 and 7 days post-infection with CVB3.
(TIF)

S6 Fig Production of cytokines by T lymphocytes from TLR3 KO mice. Representative dot plots from data presented on Fig 4A of production of IFN-γ (top panels), TNF-α (center panels), and IL-17 (bottom panels) by CD4^+^ and CD8^+^ T lymphocytes 3, 7 and 12 days post-infection with CVB3.
(TIF)

S7 Fig Heart and pancreas morphologies are not affected by deficiency on TLR3 or IL-17. Heart (top panel) and pancreas (bottom panel) sections from uninfected C57BL/6 (WT), TLR3 KO and IL-17 KO mice stained with hematoxylin and eosin.
(TIF)

S8 Fig Production of cytokines by T lymphocytes from TLR3 KO mice after CVB3 infection and DCs transfer. Representative dot plots from data presented on Fig 7A of production of IFN-γ (left panels), TNF-α (right panels), and IL-17 (bottom panels) by CD4^+^ and CD8^+^ T lymphocytes after infection with CVB3 from WT, TLR3 KO mice, or TLR3 KO mice transferred with DCs.
(TIF)

S9 Fig Production of cytokines by T lymphocytes from TLR3 KO mice after CVB3 infection and DCs transfer. Absolute number of CD4^+^ and CD8^+^ T lymphocytes producing IFN-γ (left panels), IL-17 (center panels), and TNF-α (right panels) after CVB3 infection from WT, TLR3 KO mice, or TLR3 KO mice transferred with DCs.
(TIF)
