# Supplementary figures and images for: TLR3 is required for survival following Coxsackievirus B3 infection by driving T lymphocyte activation and polarization: The role of dendritic cells
Source: PLoS One. 2017 Oct 3;12(10):e0185819. doi: 10.1371/journal.pone.0185819 (PMC5626506; doi:10.1371/journal.pone.0185819)

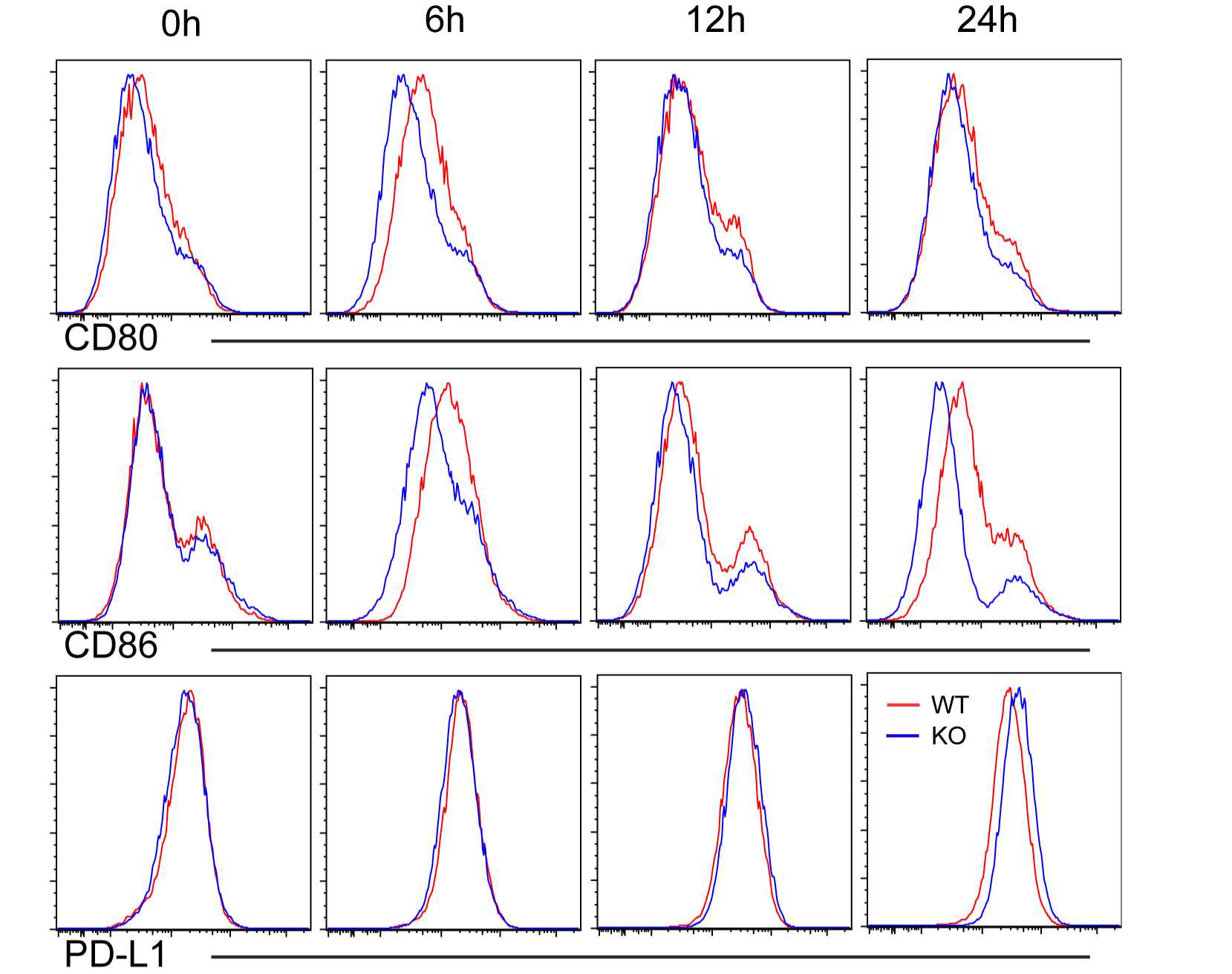

Supplement: S1 Fig — Representative histograms from data presented on Fig 1A of CD80, CD86 and PD-L1 expression by DCs 0, 6, 12 and 24 hours post-infection with CVB3. (TIF) [file pone.0185819.s001.tif]

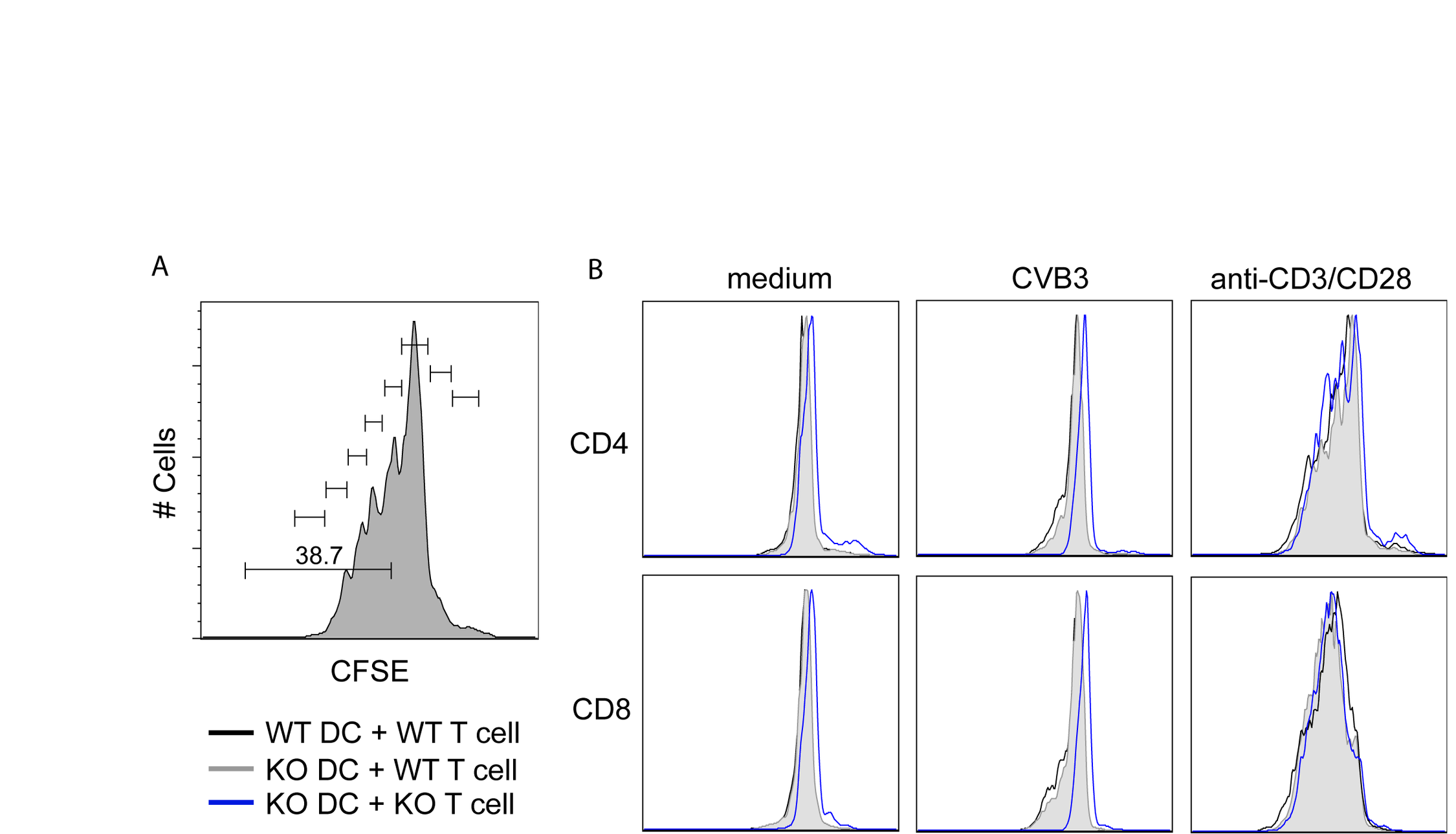

Supplement: S2 Fig — (A) Representative histogram of proliferation of T lymphocytes stimulated with anti-CD3/CD28. Bars indicate different rounds of cell division. The last rounds of division represented percentage of proliferation. (B) Representative histograms from data presented on Fig 2A of CD4+ (top panel) and CD8+ (bottom panel) T lymphocytes proliferation stimulated with DCs in the presence of medium (left), CVB3 (center), or anti-CD3/CD28 (right). (TIF) [file pone.0185819.s002.tif]

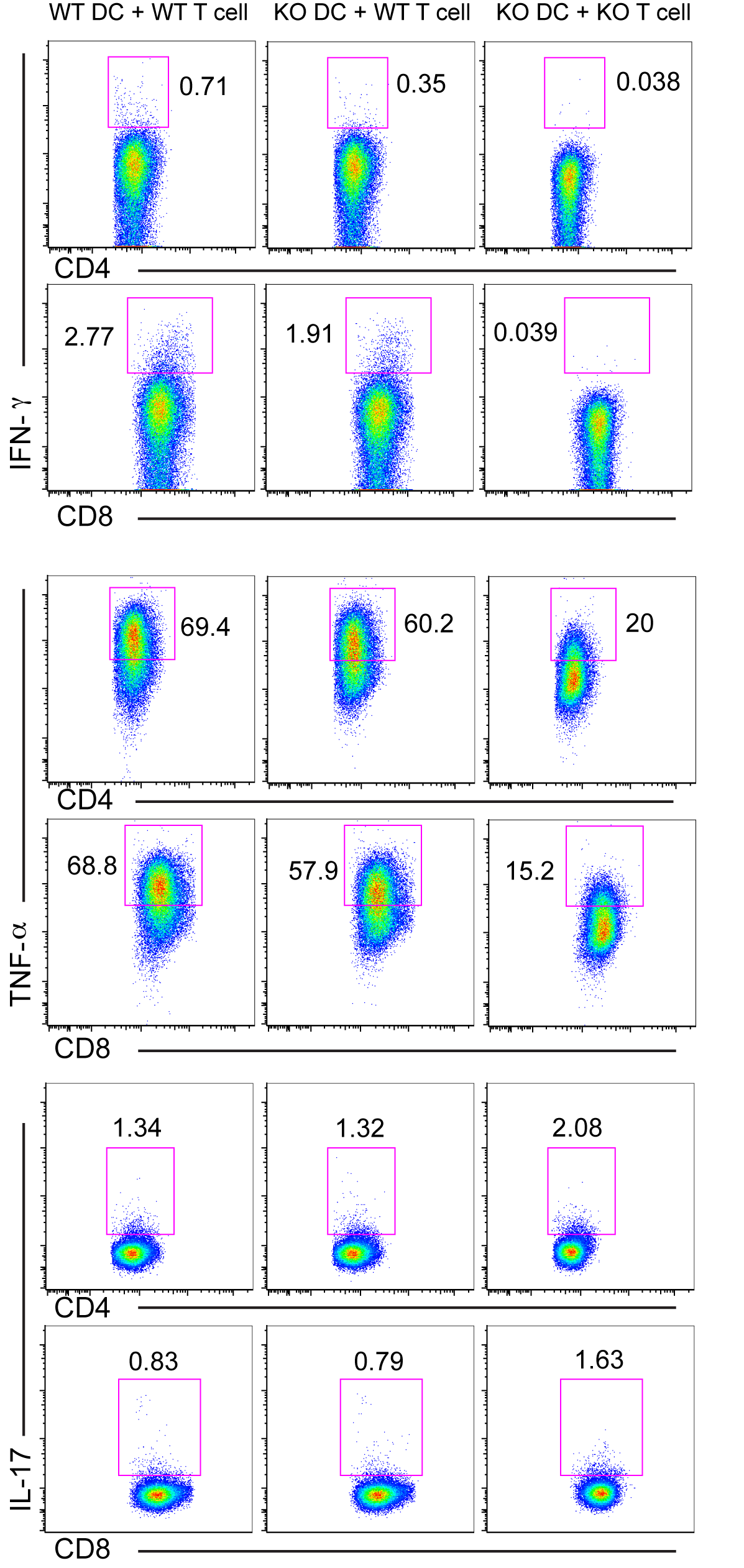

Supplement: S3 Fig — Representative dot plots from data presented on Fig 2B of production of IFN-γ (top panels), TNF-α (center panels), and IL-17 (bottom panels) by CD4+ and CD8+ T lymphocytes stimulated with DC in the presence of CVB3. (TIF) [file pone.0185819.s003.tif]

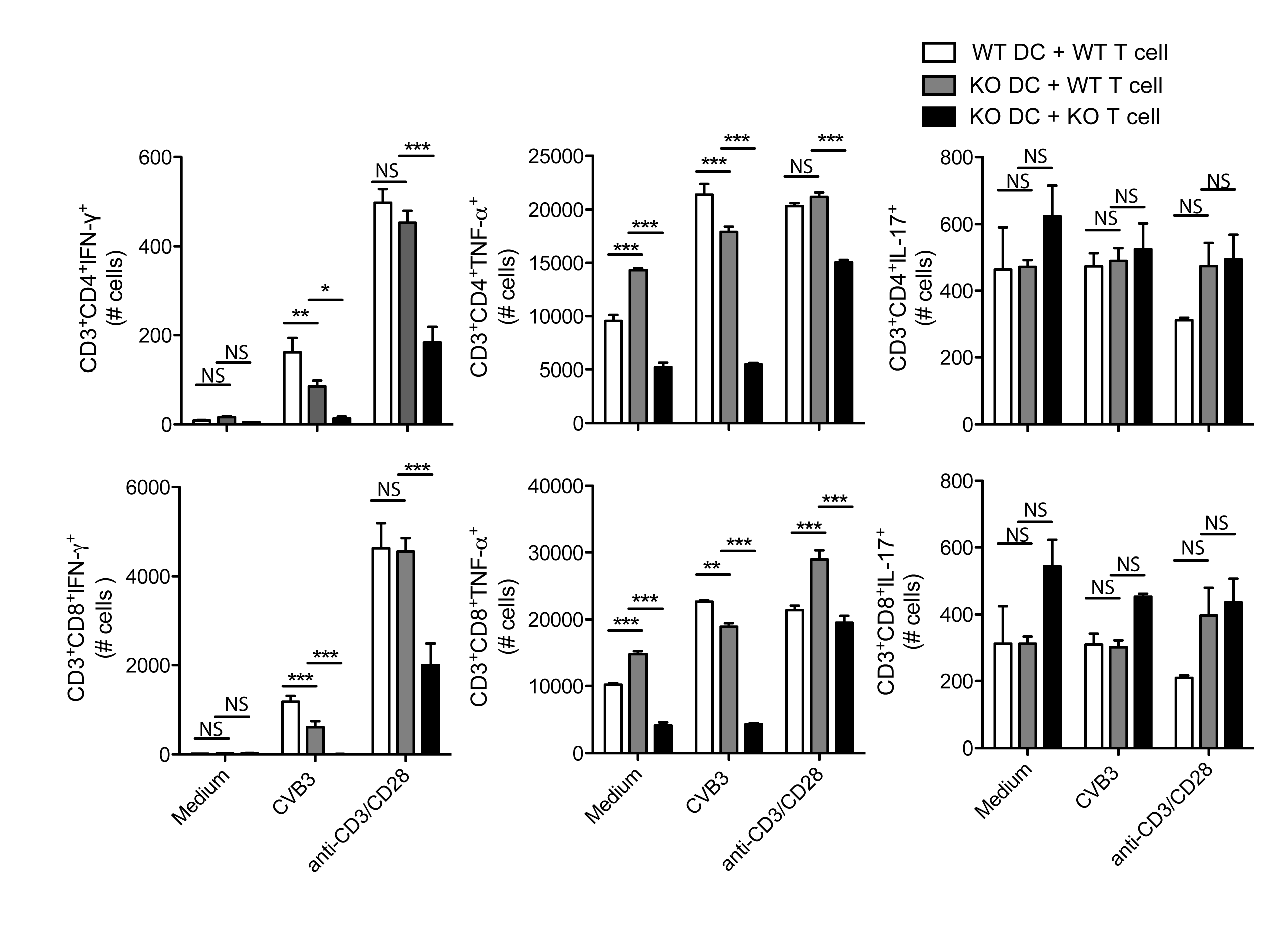

Supplement: S4 Fig — Absolute number of CD4+ and CD8+ T lymphocytes producing IFN-γ (left panels), TNF-α (center panels), and IL-17 (right panels) after stimulation with DC in the presence of medium, CVB3 or anti-CD3/CD28. All analyzed parameters in CVB3-infected condition are significantly different between WT DC + WT T cell vs KO DC + KO T cell. (TIF) [file pone.0185819.s004.tif]

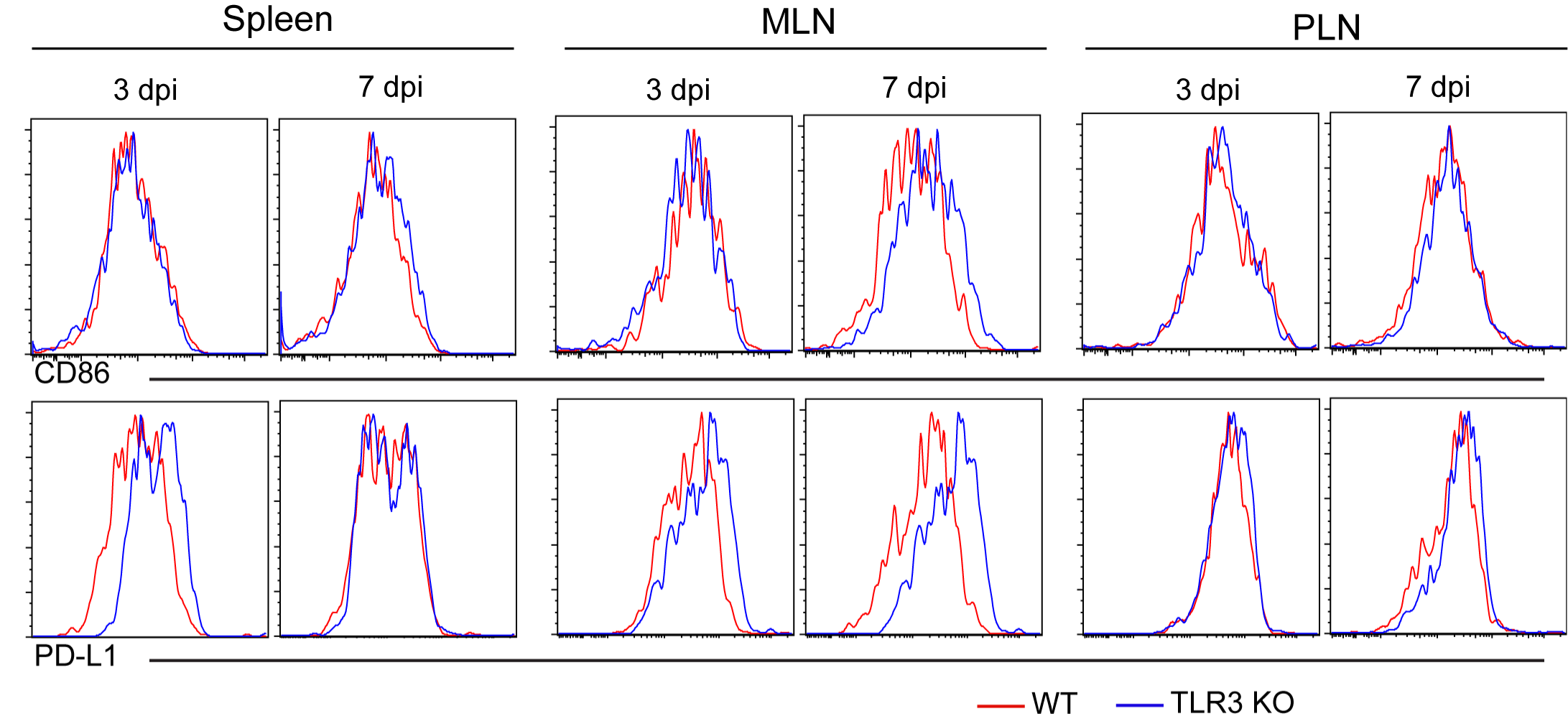

Supplement: S5 Fig — Representative histograms from data presented on Fig 3 of CD86 and PD-L1 expression by DCs on spleen, mediastinal (MLN) and pancreatic (PLN) lymph nodes 3 and 7 days post-infection with CVB3. (TIF) [file pone.0185819.s005.tif]

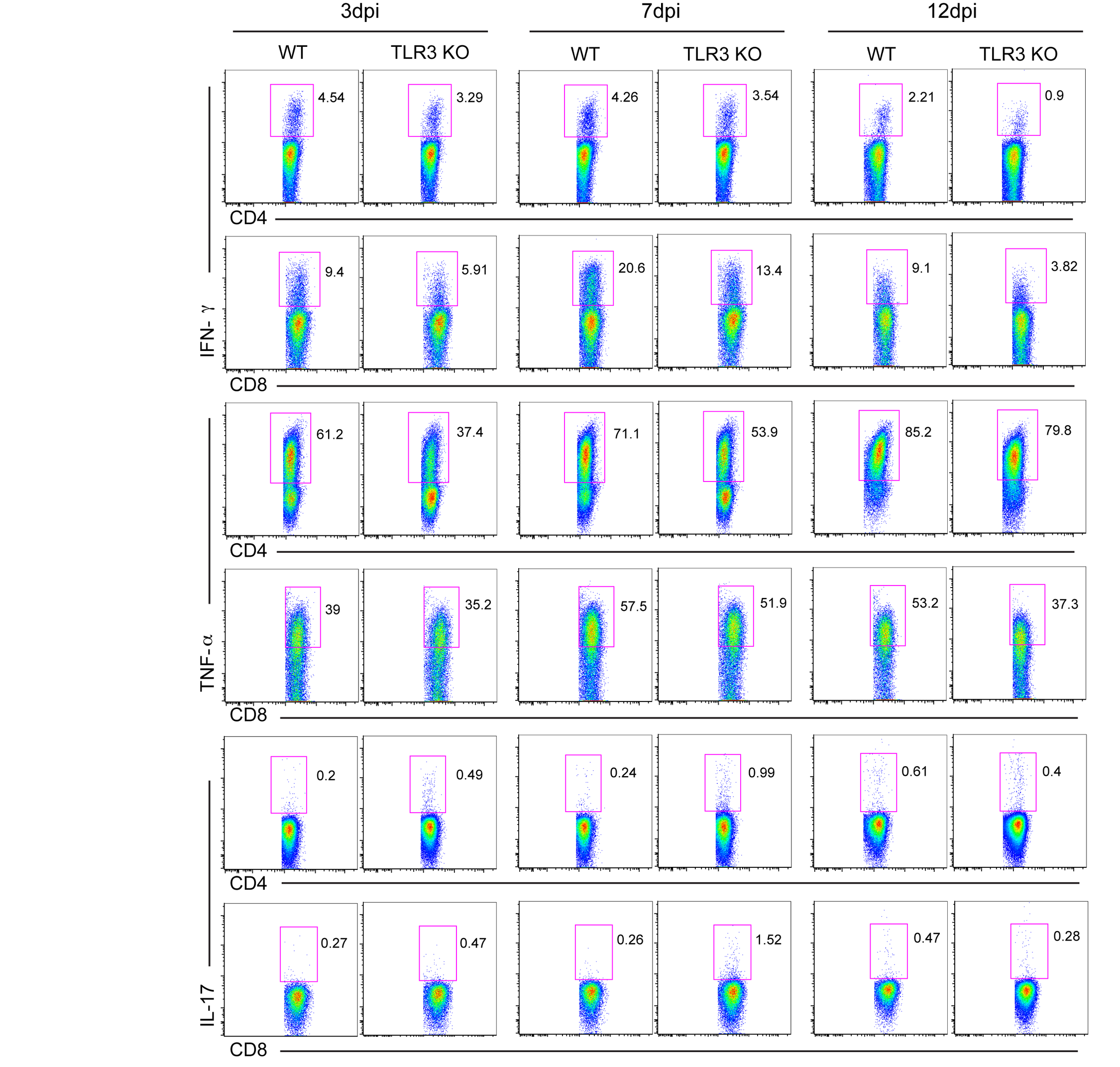

Supplement: S6 Fig — Representative dot plots from data presented on Fig 4A of production of IFN-γ (top panels), TNF-α (center panels), and IL-17 (bottom panels) by CD4+ and CD8+ T lymphocytes 3, 7 and 12 days post-infection with CVB3. (TIF) [file pone.0185819.s006.tif]

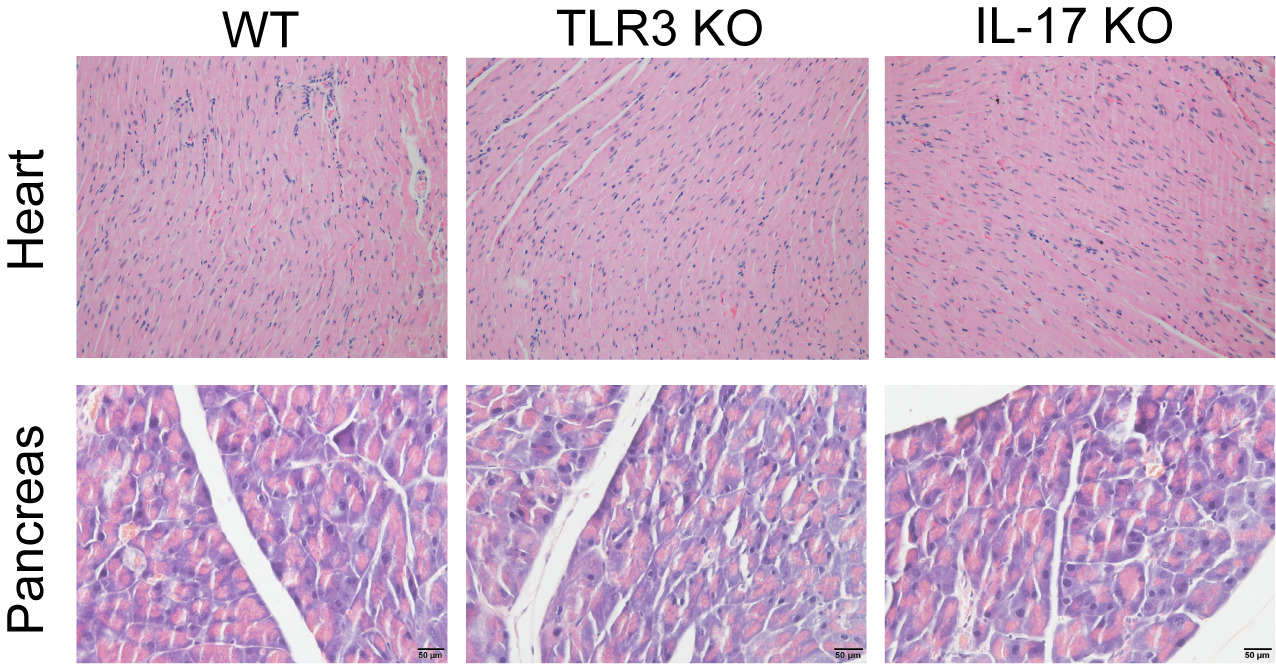

Supplement: S7 Fig — Heart (top panel) and pancreas (bottom panel) sections from uninfected C57BL/6 (WT), TLR3 KO and IL-17 KO mice stained with hematoxylin and eosin. (TIF) [file pone.0185819.s007.tif]

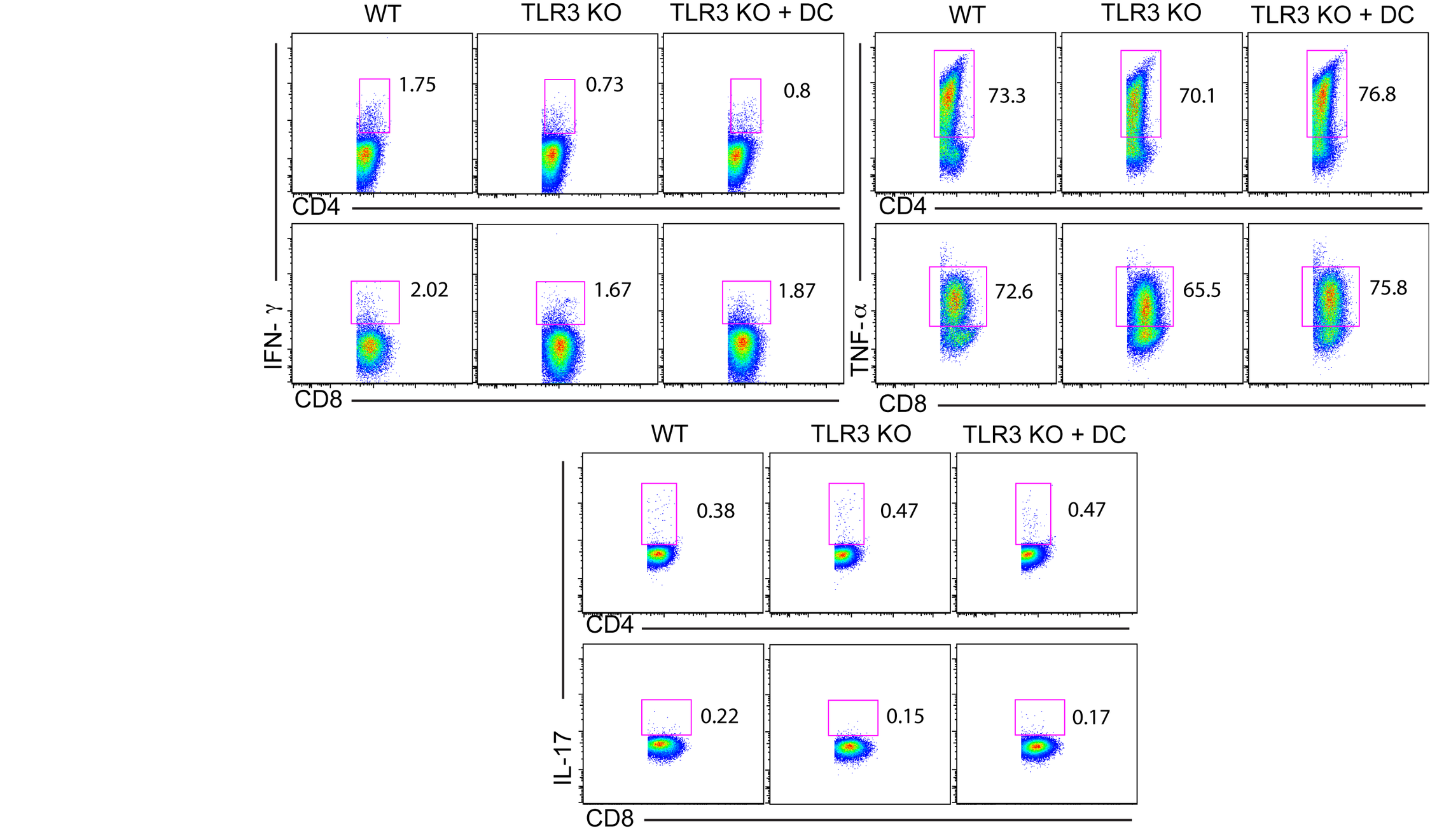

Supplement: S8 Fig — Representative dot plots from data presented on Fig 7A of production of IFN-γ (left panels), TNF-α (right panels), and IL-17 (bottom panels) by CD4+ and CD8+ T lymphocytes after infection with CVB3 from WT, TLR3 KO mice, or TLR3 KO mice transferred with DCs. (TIF) [file pone.0185819.s008.tif]

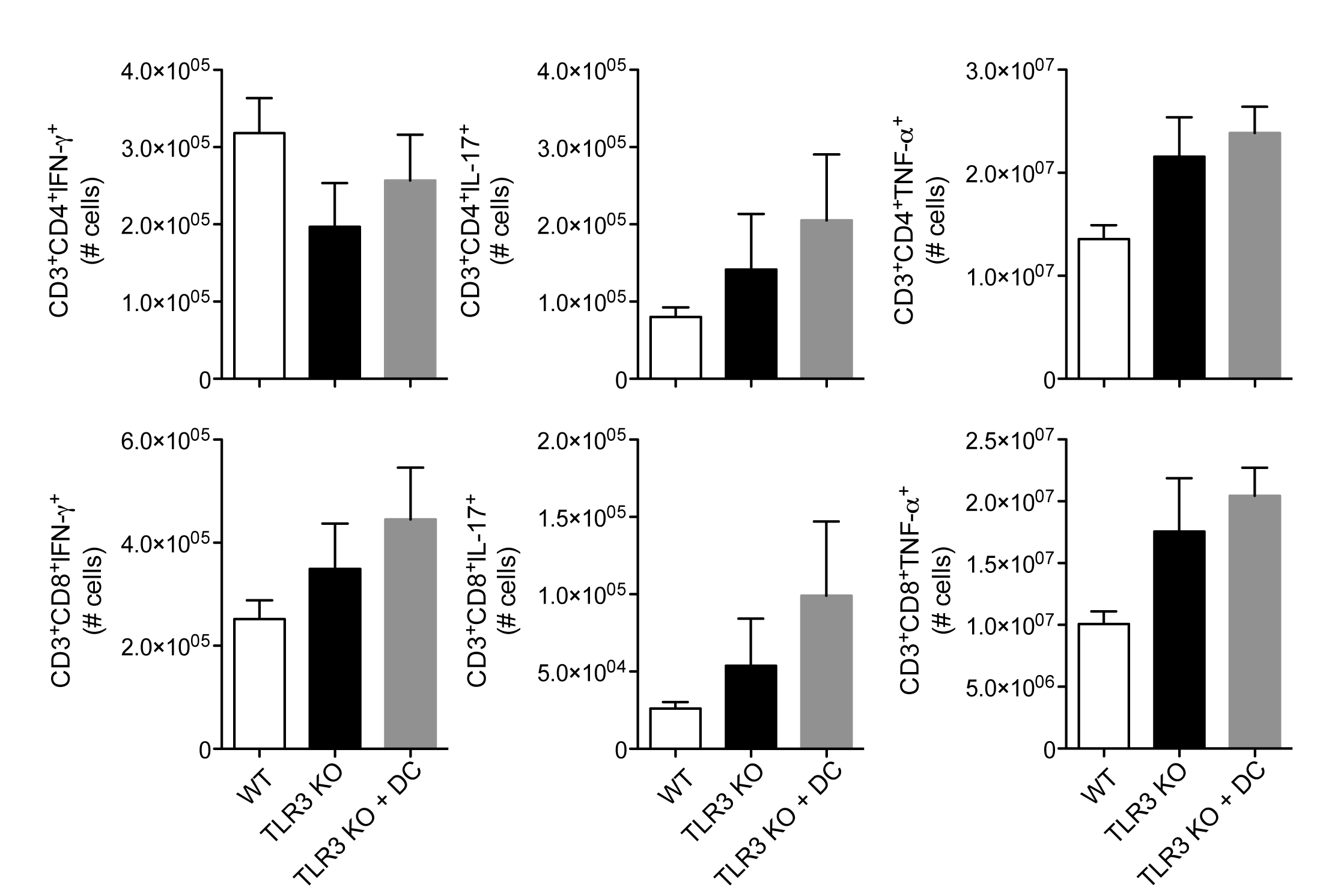

Supplement: S9 Fig — Absolute number of CD4+ and CD8+ T lymphocytes producing IFN-γ (left panels), IL-17 (center panels), and TNF-α (right panels) after CVB3 infection from WT, TLR3 KO mice, or TLR3 KO mice transferred with DCs. (TIF) [file pone.0185819.s009.tif]
